# Cystic lymph node metastases of squamous cell carcinoma of Waldeyer's ring origin

**DOI:** 10.1038/sj.bjc.6690229

**Published:** 1999-03

**Authors:** S Regauer, S Mannweiler, W Anderhuber, A Gotschuli, A Berghold, J Schachenreiter, R Jakse, A Beham

**Affiliations:** Department of Pathology, University of Graz, Auenbruggerplatz 25, Graz, A-8036 Graz, Austria;; Department of General ORL, University of Graz, Auenbruggerplatz 25, Graz, A-8036 Graz, Austria;; Institute of Medical Statistics, Informatics and Documentation, University of Graz, Engelgasse 13/1, Graz, A-8010 Graz, Austria; Department of ORL, Elisabethinen Hospital, Elisabethinergasse 14, Graz, A-8020 Graz, Austria

**Keywords:** Waldeyer's ring, oropharynx, squamous cell carcinoma, cystic metastases, branchiogenic carcinoma, neck cysts

## Abstract

We analysed in a retrospective study the frequency of cystic lymph node (LN) metastases in neck dissection specimens of 123 patients with primary squamous cell carcinoma (SCC) arising in the palatine tonsils (62 M/14 F), the base of the tongue (38 M/5 F) and the nasopharynx (2 M/2 F). Eighty-two per cent of patients had metastases (64 tonsillar SCC, 33 base of tongue SCC and all four nasopharynx SCC) in 368 LN of a total 2298 sampled LN. Thirty-nine per cent of patients had exclusively solid metastases and 37% of patients had exclusively cystic metastases. A total of 62 patients had some signs of cyst formation in one or more metastatically affected LN (27 with only histological evidence of cyst formation with luminal diameters < 5 mm, 35 with clinically detectable cyst with luminal diameter > 5 mm). Cystic metastases were more common in patients with SCC of the base of the tongue (*P* = 0.005), while solitary clinically evident cystic metastasis with lumina > 5 mm were found exclusively in tonsillar carcinoma (*P* = 0.024). In comparison with solid metastases, cyst formation was associated with N-categories (N2b and N3, *P* = 0.005) in SCC of the base of the tongue origin. No such association was observed for tonsillar SCC (*P* = 0.65). The primary mechanism of cyst formation was cystic degeneration. © 1999 Cancer Research Campaign


*British Journal of Cancer* (1999) **79**(9/10), 1437–1442
© 1999 Cancer Research Campaign
Article no. bjoc.1998.0229

### Cystic lymph node metastases of squamous cell carcinoma of Waldeyer's ring origin

**S Regauer[1], S Mannweiler[1], W Anderhuber[2], A Gotschuli[2], A Berghold[3], J Schachenreiter[4], R Jakse[4] and A Beham[1]**

Departments of [1]Pathology and [2]General ORL, University of Graz, Auenbruggerplatz 25, A-8036 Graz, Austria; [3]Institute of Medical Statistics, Informatics and Documentation, Engelgasse 13/1, University of Graz A-8010 Graz, Austria; and [4]Department of ORL, Elisabethinen Hospital, Elisabethinergasse 14, A-8020 Graz, Austria

**Summary** We analysed in a retrospective study the frequency of cystic lymph node (LN) metastases in neck dissection specimens of 123 patients with primary squamous cell carcinoma (SCC) arising in the palatine tonsils (62 M/14 F), the base of the tongue (38 M/5 F) and the nasopharynx (2 M/2 F). Eighty-two per cent of patients had metastases (64 tonsillar SCC, 33 base of tongue SCC and all four nasopharynx SCC) in 368 LN of a total 2298 sampled LN. Thirty-nine per cent of patients had exclusively solid metastases and 37% of patients had exclusively cystic metastases. A total of 62 patients had some signs of cyst formation in one or more metastatically affected LN (27 with only histological evidence of cyst formation with luminal diameters < 5 mm, 35 with clinically detectable cyst with luminal diameter > 5 mm). Cystic metastases were more common in patients with SCC of the base of the tongue ($P = 0.005$), while solitary clinically evident cystic metastasis with lumina > 5 mm were found exclusively in tonsillar carcinoma ($P = 0.024$). In comparison with solid metastases, cyst formation was associated with N-categories (N2b and N3, $P = 0.005$) in SCC of the base of the tongue origin. No such association was observed for tonsillar SCC ($P = 0.65$). The primary mechanism of cyst formation was cystic degeneration.

**Keywords:** Waldeyer's ring; oropharynx; squamous cell carcinoma; cystic metastases; branchiogenic carcinoma; neck cysts

Carcinomas of the oro/nasopharynx often present with regional lymph node (LN) metastases before the primary tumour is discovered. Some of the cervical LN metastases feature prominent cyst formation. Solitary cystic LN metastases have often been mistaken for primary squamous cell carcinomas (SCC) originating within a branchiogenic lateral neck cyst (LNC), resulting in the commonly used terms 'branchioma' and 'branchiogenic carcinoma' (von Volkmann, 1882; Wolff et al, 1979; Khafif et al, 1989; Parks and Karmody, 1992; Carroll et al, 1993; Hall and Dexter, 1993; Flanagan et al, 1994; Knobber et al, 1995). For patients with an isolated finding of SCC in a neck LN, a search for a primary carcinoma in the upper aerodigestive tract is mandatory, including US, MRI and CT, as well as extensive biopsies of the base of tongue, nasopharynx and tonsillectomies in the case of clinically occult tumours (Spiro et al, 1983; Flanagan et al, 1994). This clinical practice has provided overwhelming evidence in recent years that isolated cystic SCC in neck LN are metastases from primary SCC of the palatine tonsils, the base of the tongue and the nasopharynx, tissues also collectively referred to as the Waldeyer's ring (Micheau et al, 1974, 1990; Flanagan et al, 1994; Thompson and Heffner, 1998). Although this explanation has been slow to gain wide recognition and claims of the first true 'branchiogenic carcinoma' have been made rather recently (Micheau et al, 1974; Jones et al, 1993), cystic SCC in cervical LN is now regarded as a typical presentation of metastatic SCC arising in the oro/nasopharynx. At the same time, the idea of a primary branchiogenic carcinoma has become a vanishing concept (Thompson and Heffner, 1998). Despite the body of literature about this topic, there is little information regarding the frequency of cystic LN metastases from SCC of the Waldeyer's ring. Our goal was to establish the frequency of cystic LN metastases in our series of 123 patients with SCC of Waldeyer's ring origin who had been treated primarily surgically along with neck dissections. Furthermore, we discuss the morphological features and clues for establishing a diagnosis of cystic LN metastases rather than 'branchiogenic carcinoma'.

*Received 2 March 1998*
*Revised 4 September 1998*
*Accepted 7 October 1998*

*Correspondence to:* S Regauer

### MATERIALS AND METHODS

Between 1984 and 1997, 108 patients with biopsy-proven SCC of Waldeyer's ring origin underwent neck dissections at the ORL Department of the University Hospital in Graz, Austria. Fifteen patients were treated at the Elisabethinen Hospital, Graz, Austria, between 1992 and 1997. Archival formalin-fixed, paraffin-embedded haematoxylin and eosin (HE) stained sections of all primary SCC and their corresponding neck dissections were reviewed. SCC were separated into (1) tonsillar origin (palatine tonsil), (2) base of tongue origin and (3) nasopharyngeal origin. Extensive tumours involving the entire oropharynx, and multiple or synchronous intraoral carcinomas were not included in this study, because we were not able to determine the exact origin of the SCC in these cases. Initial diagnosis was established by biopsy and followed by definitive surgical treatment of the primary SCC in all but two patients. All surgically removed tumours and neck dissections were classified histologically according to the most recent TNM classification (Sobin and Wittekind, 1997). All carcinomas were graded from well-differentiated keratinized, G1; moderately differentiated, focally keratinized, G2; poorly differentiated non-keratinized, G3; and undifferentiated, G4. The neck dissections were either classical radical, extended radical, modified radical type I (preserving selectively the accessory nerve), modified radical type II (preserving accessory nerve and sternocleidomastoid muscle), modified radical type III (preserving spinal accessory nerve, internal jugular vein and sternocleidomastoid muscle) (Shah, 1996). Except for two patients, the neck dissections were performed unilaterally. The LN metastases were divided into solid and cystic. The luminal diameter of cystic metastases was determined on the HE-stained histological sections with a graded ocular and micrometer scale. Ultrasound examination (small parts sonography, Toshiba, 75 MHz transducer) was the method of choice for preoperative screening for LN metastases, which has been assigned the 'certainty factor' of C2 in the TNM-classification. In our institutions, LN greater than 5 mm in the accessory region of the neck and greater than 9 mm in the mandibular region were considered suspicious for metastases. With ultrasound, we were able to visualize cystic areas within a suspicious LN with a luminal diameter greater than 5 mm. Cystic lumina greater than 5 mm were therefore considered clinically detectable and microscopically evident lumina less than 5 mm were considered clinically occult. Taking into account the preoperative technical possibilities and clinical limitations of recognizing cystic metastases with lumina less than 5 mm, we divided the cystic metastases in our patient pool into two categories: occult (with cystic lumina less than 5 mm) and clinically evident (with luminal diameters greater than 5 mm). Furthermore, histologically verified solitary cystic LN metastases were recorded separately, as their clinical relevance especially in reference to primary branchiogenic carcinoma needs to be discussed.

Correlation of cyst formation and (a) extent of primary tumour = T-stage; (b) histological grade = G of primary tumours and metastases; and (c) LN category was examined. Differences in distribution of cystic metastases from SCC of palatine tonsil and base of tongue were documented. Statistical analysis was based on patient numbers and the comparison of solid and cystic metastases, independent of luminal diameter. For all analyses, a chi-squared test was applied. Statistical analysis of our four nasopharyngeal SCC was not performed due to insufficient numbers of patients.

### RESULTS

### Frequency of regional LN metastases

We reviewed a total of 123 patients with primary SCC of Waldeyer's ring origin. Seventy-six SCC arose in the palatine tonsil, 43 in the base of tongue and four in the nasopharynx. Twenty-two patients had a histologically verified 'N0 neck'. Metastases were identified in 101 (82%) patients, 64 patients with tonsillar SCC, 33 patients with base of tongue SCC and all four patients with nasopharyngeal carcinomas (see Table 1). Thirty-nine patients had exclusively solid metastases, 37 patients had exclusively cystic metastases and 25 patients had both cystic and solid LN metastases. The luminal diameters of the cystic metastases ranged from minimally 1 mm to maximally 20 mm. Of the 37 patients with exclusively cystic metastases, only 23 had clinically detectable cystic metastases with luminal diameters greater than 5 mm. A total of 62 patients (61.3%) had histological evidence of cyst formation in one or more LN metastases, either with or without concomitant solid LN metastases (36 patients with palatine tonsillar SCC, 24 patients with base of tongue SCC and two patients with nasopharynx SCC). Clinically occult cystic metastases (< 5 mm) were detected in 27/62 patients, clinically detectable cystic metastases (> 5 mm) were observed in 35 patients (see Table 2). Multiple cystic LN metastases were more common in patients with SCC of base of tongue (19/33 patients = 57.5%) than in patients with tonsillar SCC (18/64 patients = 28%; $P = 0.005$). The distribution of cystic, solid and mixed metastases with reference to T-stage and N-category is shown in Figure 1A–C and Figure 2A–C. A comparison of cystic and solid

**Table 1** Distribution of SCC of Waldeyer's ring origin and frequency of regional metastatic disease ($n$ = 123 patients)

| | Palatine tonsil | Base of tongue | Nasopharynx | Total patients |
|---|---|---|---|---|
| Total patients | 76 (100%) | 43 (100%) | 4 (100%) | 123 (100%) |
| Mean age (years) | 59.7 | 55.6 | 50.5 | |
| Male | 62 (81.6%) | 38 (88.6%) | 2 (50%) | 102 (83%) |
| Female | 14 (18.4%) | 5 (11.4%) | 2 (50%) | 21 (17.0%) |
| Patients without LN metastases, pN0 | 12 (15.7%) | 10 (23.2%) | 0 (0%) | 22 (17.8%) |
| Patients with LN-metastases | 64 (84.3%) | 33 (76.8%) | 4 (100%) | 101 (82.2%) |
| Male | 52 | 28 | 2 | 82 |
| Female | 12 | 5 | 2 | 19 |

**Table 2** Regional lymph node metastases in patients with SCC of Waldeyer's ring ($n$ = 101 patients)

| | Palatine tonsil | Base of tongue | Nasopharynx | Total ($n$ = 101) |
|---|---|---|---|---|
| Patients | 64 (100%) | 33 (100%) | 4 (100%) | 101 (100%) |
| Only solid LN metastases | 28 (43.7%) | 9 (27.3%) | 2 (50%) | 39 (38.6%) |
| Only cystic LN metastases | 21 (32.8%) | 15 (45.4%) | 1 (25%) | 37 (36.6%) |
| Luminal diameter < 5 mm (clinically occult) | 7 (10.9%) | 7 (21.2%) | 0 (0%) | 14 (13.8%) |
| Luminal diameter > 5 mm (clinically detectable) | 14 (21.8%) | 8 (24.2%) | 1 (25%) | 23 (22.7%) |
| Solid and cystic metastases | 15 (23.4%) | 9 (27.2%) | 1 (25%) | 25 (24.76%) |
| Cystic LN metastases (any diameter with or without concomitant solid metastases) | 36 (56.2%) | 24 (72.7%) | 2 (50%) | 62 (61.3%) |
| Luminal diameter < 5 mm (clinically occult) | 17 (26.6%) | 9 (27.2%) | 1 (25%) | 27 (26.7%) |
| Luminal diameter > 5 mm (clinically detectable) | 19 (29.6%) | 15 (45.4%) | 1 (25%) | 35 (34.6%) |

 © Cancer Research Campaign 1999

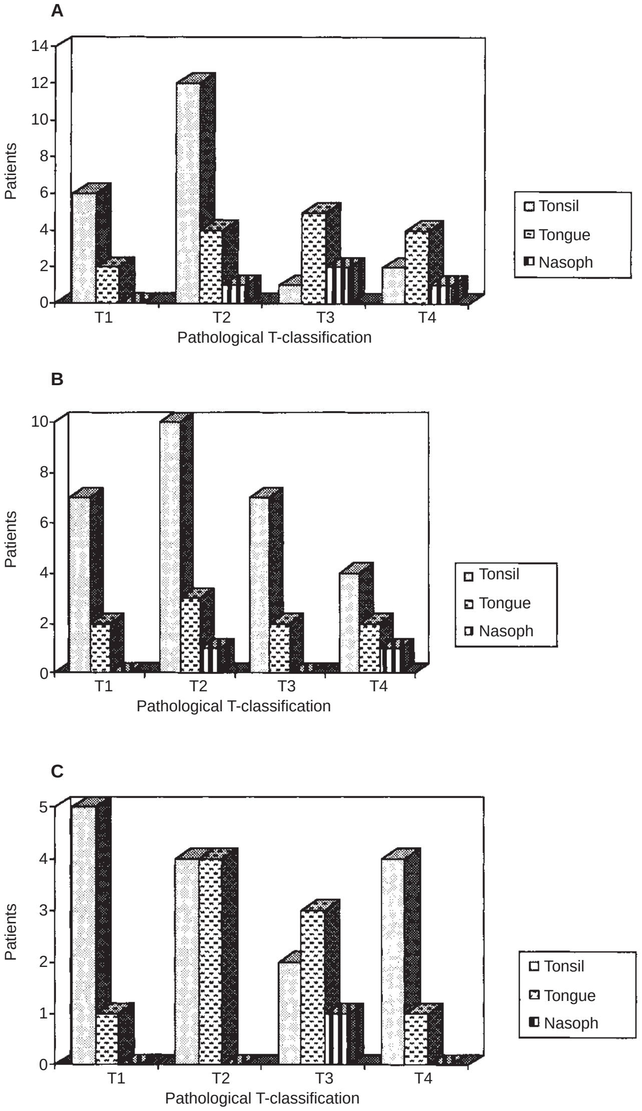

**Figure 1** (**A**) Cyst formation and T-classification ($n$ = 37 patients), (**B**) solid metastases and T-classification ($n$ = 39 patients), and (**C**) mixed (solid and cystic) metastases and T-classification ($n$ = 25 patients)

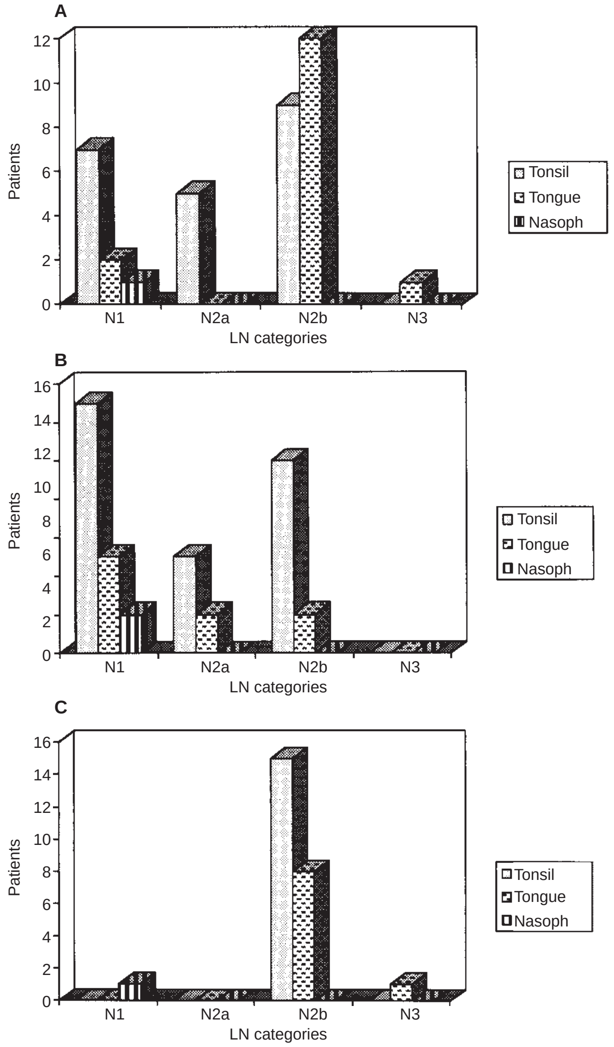

**Figure 2** (**A**) Cyst formation and lymph node status ($n$ = 37 patients), (**B**) solid metastases and lymph node status ($n$ = 39 patients), and (**C**) mixed (solid and cystic) metastases and lymph node status ($n$ = 25 patients)

metastases showed a significant association of cyst formation and N-category in SCC arising in the base of the tongue ($P$ = 0.005), but no such association was observed for SCC arising in the palatine tonsil ($P$ = 0.65). In other words, solid metastases were found more frequently with the N1-category, while cystic metastases were associated with N2b and N3 categories.

Solitary LN metastases (see Table 3) were identified in 38/101 patients, 21 of which had solid metastases. The 17 solitary cystic metastases were observed in 14/64 patients with SCC of palatine tonsillar origin and in 3/33 patients with SCC of base of tongue origin. The clinically detectable solitary cystic metastasis > 5 mm were observed exclusively in patients with tonsillar SCC ($P$ = 0.024). No solitary cystic metastasis was observed in SCC of the nasopharynx. Two patients presented with an isolated finding of a cystic mass 6 weeks before the primary tumour was detected in the tonsillectomy specimens. The other seven patients presented with a simultaneous primary SCC which was detected by clinical investigations.

A total of 2298 LN were identified in the neck dissection specimens of the 101 patients with metastatic disease and histologically examined (see Table 4). Metastases were identified in 368 LN (16% of sampled LN). Cyst formation within metastases were found in exactly 50% of all metastatically affected LN. However, cystic metastases with luminal diameters > 5 mm were identified in only 26% of all metastatically affected LN. The primary carcinomas producing cystic LN metastases were found in the palatine tonsils (20.8%), the base of the tongue (27.2%) and the nasopharynx (1.8%).

### Histology of cystic LN metastases

The majority of LN metastases were multicystic. Cavities filled with debris (Figure 3A and C) often had multiple irregular complex lumina which were lined by polypoid and papillary malignant epithelium with high mitotic rates and incomplete keratinization. The growth pattern was often endophytic, in which the

© Cancer Research Campaign 1999

**Table 3** Solitary LN metastasis in patients with SCC of Waldeyer's ring ($n$ = 101 patients)

| | Palatine tonsil | Base of tongue | Nasopharynx | Total LN |
|---|---|---|---|---|
| Total patients with LN metastases | 64 (100%) | 33 (100%) | 4 (100%) | 101 (100%) |
| Solitary metastases | 28 (43.7%) | 10 (30.3%) | 0 (0%) | 38 (37.6%) |
| Solid solitary LN metastasis | 14 (21.8%) | 7 (21.2%) | 0 (0%) | 21 (20.7%) |
| Cystic solitary LN metastasis luminal diameter < 5 mm | 5 (7.8%) | 3 (9%) | 0 (0%) | 8 (16.8%) |
| Cystic solitary LN metastasis luminal diameter > 5 mm | 9 (14%) | 0 (0%) | 0 (0%) | 9 (14%) |

**Table 4** Metastases in regional lymph nodes (LN) from SCC of Waldeyer's ring origin ($n$ = 368 LN)

| | Palatine tonsil | Base of tongue | Nasopharynx | Total LN |
|---|---|---|---|---|
| Total LN sampled | 1349 | 878 | 71 | 2298 |
| Metastatically affected lymph nodes (total) | 179 | 170 | 19 | 368 (100%) |
| Cystic metastases (total) with any diameter | 77 (43%) | 101 (60%) | 7 (37%) | 185 (50%) |
| Cystic metastases with luminal diameter < 5 mm | 35 (19%) | 49 (29%) | 5 (26%) | 89 (24%) |
| Cystic metastases with luminal diameter > 5 mm | 42 (23%) | 52 (31%) | 2 (11%) | 96 (26%) |
| Solid metastases | 102 (57%) | 69 (40%) | 12 (63%) | 183 (50%) |

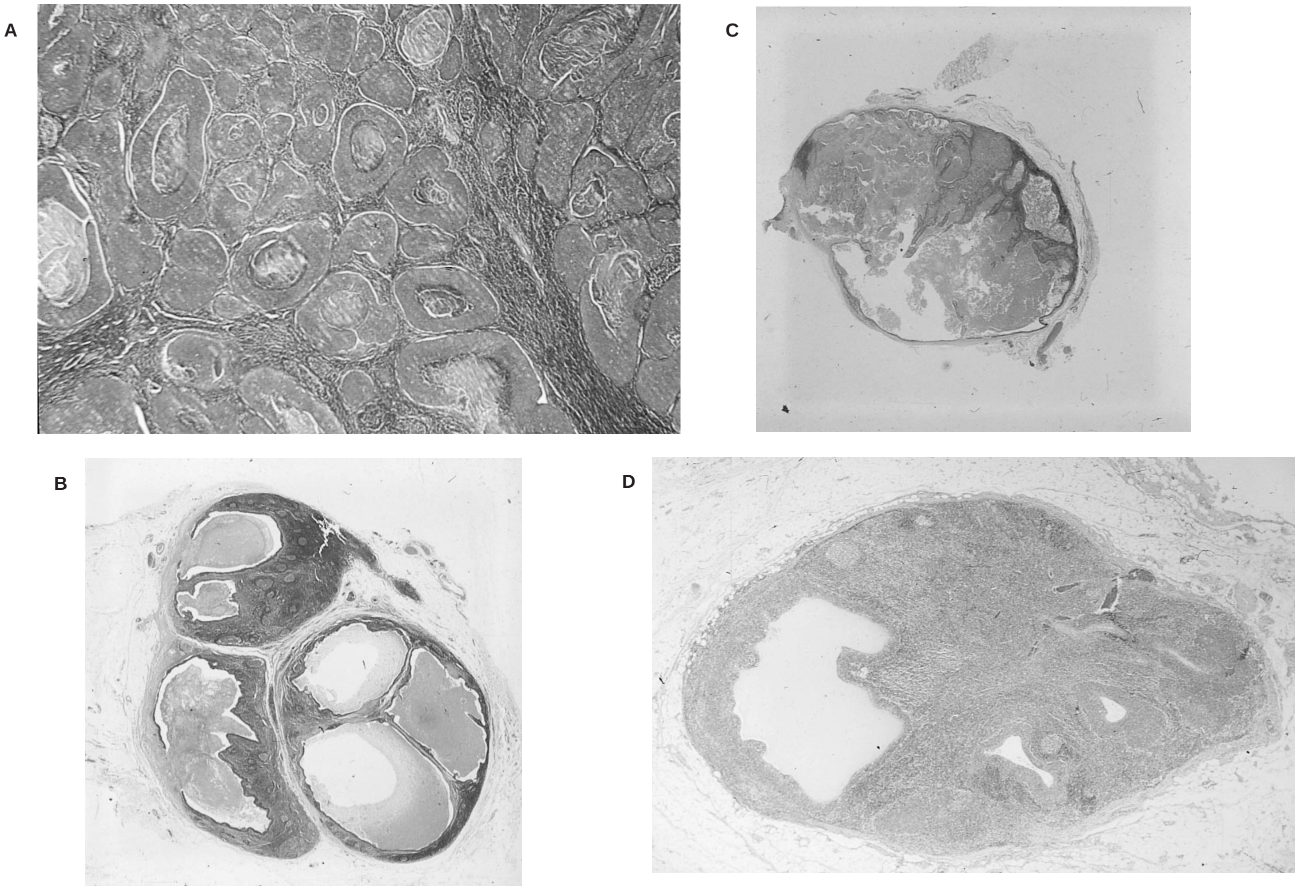

**Figure 3** (**A**) LN metastases of a SCC of the base of the tongue with multiple 1–2 mm in diameter, debris-filled cysts, ×16.5. (**D**) Cystic fluid-filled LN metastases of a tonsillar SCC with luminal diameter of 2 mm in a 6.5mm large lymph node, ×12.5. (**C**) Clinically detectable cystic metastases of a base of tongue SCC, luminal diameter of 12 mm, filled with keratin squames and debris, with irregular papillary epithelial proliferation, ×1.25. (**B**) Multicystic, clinically evident, fluid-filled metastases of a tonsillar SCC, ×1.25

lining of the cystic spaces grew downward into the lymphoid tissues. A minority of cystic metastases contained granular and/or homogenous eosinophilic fluid (Figure 3B and D) intermixed with occasional neutrophilic granulocytes. The fluid-filled cavities had smooth and regular outlines (Figure 3B). The majority of metastastic SCC was bland, recapitulating the transitional type epithelium of tonsillar crypts and was graded as moderately to poorly differentiated. A range of differentiation from moderately

 *© Cancer Research Campaign 1999*

differentiated SCC with focal para- or dyskeratotic squamous pearls (G2) to widely invasive, poorly differentiated (G3) and rarely undifferentiated (G4) carcinoma was observed. No well-differentiated (G1) carcinoma was identified. The histological grade of the primary tumour and metastases was essentially identical. Of the 185 cystic metastases, 144 contained cellular debris, necrotic keratinocytes (Figure 3A and C), 37/185 LN contained homogenous eosinophilic fluid (Figure 3B and D) and four were empty.

Distant haematogenous metastases from oro/nasopharyngeal SCC were observed in 12/123 patients with primary SCC of Waldeyer's ring origin. Three patients had cystic metastases to lung, skin and bone, which were large metastases and the cystic character was clinically evident. The other metastases (skin, lung and bone) were solid.

### DISCUSSION

We present the first detailed study on the frequency of cystic metastases in 101 patients having metastatic disease from SCC of Waldeyer's ring. We demonstrate a rather high rate of cyst formation in cervical LN metastases (61% of patients), however, clinically detectable cystic metastases with a luminal diameter > 5 mm were less common (in one-third of patients and 26% of metastatic LN). Cystic metastases in general had a higher frequency in base of tongue SCC than in tonsillar SCC, while clinically detectable solitary cystic metastases were identified in only nine patients and exclusively in tonsillar SCC.

Solitary cystic LN metastases, in the absence of a primary tumour, in particular, raise the question of a primary branchiogenic carcinoma. Considering the rarity of isolated cervical cysts with SCC, they have received overwhelming attention in the literature due to the clinical implication of a mistaken diagnosis of a primary branchiogenic carcinoma. The evolution of in-situ or invasive carcinoma from non-neoplastic squamous epithelium through dysplasia has been viewed as the most important criterion for the histopathological diagnosis of a primary branchiogenic carcinoma. We did not observe a transition from nontumorous regularly stratified squamous epithelium to carcinoma in our series of LNC (Regauer et al, 1997) or in this study of cystic metastases, in which the entire squamous epithelial lining demonstrated malignant cytological features with moderate or poor differentiation. Solitary LN metastasis with well-differentiated (G1) SCC, which would most closely resemble epithelium of a benign LNC, did not occur in our series.

We only had two patients who initially presented with an isolated solitary cystic carcinoma. Their primary SCC were identified within 6 weeks. During the last 5 years, we had not had a single case of an isolated solitary cystic carcinoma without an immediately evident primary carcinoma, contrary to the literature which suggests the existence of a true branchiogenic carcinoma (Spiro et al, 1983; Browder et al, 1984; Shreedhar and Tooley, 1984; Parks and Karmody, 1992; Jacobsen et al, 1998; Redaelli de Zinis et al, 1998). One of these SCC, however, was extremely well differentiated and arose from a background of longstanding chronic inflammation and scarring (Shreedhar and Tooley, 1984), another arose from preauricular ectodermal remnants of the first branchial cleft (Parks and Karmody, 1992). We did not observe mucoepidermoid differentiation or mucus-containing cells, either in our series of benign lateral neck cysts (Regauer et al, 1997) or in this series of cystic carcinomas which contrasts a report of a primary well-differentiated mucoepidermoid branchiogenic carcinoma (Browder et al, 1984). Primary cystic carcinoma in an LN or carcinoma arising in a branchiogenic cyst is probably a hypothetical entity, or at least an extremely rare event. Lymphocytes around a primary carcinoma may be responsible for the silent presentation of the primary SCC or the occurrence of occult SCC, as tumour-infiltrating lymphocytes and a marked inflammatory response have been associated with host responses resulting in local tumour growth control such as regression and complete involution, e.g. in malignant melanoma (Clark et al, 1989; Barr, 1994) or head and neck tumours (Sessions and Hudkins, 1993). Partial or complete immunological regression (disappearance of the primary tumour) could serve as a plausible explanation for some so-called 'branchiogenic' carcinomas without a detectable primary SCC even after many years of follow-up (Martin et al, 1950; Thompson and Heffner, 1998).

Collection of cellular debris and cystic degeneration was the mechanism of cyst formation in the majority of cysts. Other mechanisms may be imperative, however, as some cysts were filled with eosinophilic fluid. The mechanism of cyst formation in these cases remains elusive. The fluid content could implicate an osmotic/secretory activity, or an active transport mechanism across the epithelium as seen in salivary gland epithelium, possibly through displaced salivary gland cells. The observation of cystic LN metastases derived from oro/nasopharyngeal SCC will remain an unexplained, site-specific phenomenon, although distant metastases from oro/nasopharyngeal SCC to lung, skin and bone were cystic in 3/12 of our patients with distant haematogenous metastases. These observations speak against the theory that a special lymphatic environment induces cyst formation, and also SCC arising in other locations does not produce cystic LN metastases. The primary SCC arising in the Waldeyer's ring often demonstrates cystic spaces, especially deep inside crypts, and the cystic metastases appear to simulate the growth behaviour and growth pattern of the parent cell. We speculate that induction of cyst formation in lymphatic and haematogenous metastases is an intrinsic property of these keratinocytes that can be expressed and maintained in a permissive environment in the absence of lymphoid tissue.

In summary, SCC of Waldeyer's ring origin has a high incidence of clinically occult cystic LN metastases, but only a small percentage of clinically evident cystic metastases. Cystic LN metastases were more common in SCC of base of tongue, while clinically evident solitary cystic metastasis occurred exclusively in tonsillar SCC. In an extremely small percentage (2%) of patients, a solitary cystic metastasis was the first clinical presentation. Poor histological differentiation and the absence of transitions from benign epithelium to malignant carcinoma in the LN metastases are indicators for metastases of SCC of Waldeyer's ring origin rather than a primary 'branchiogenic carcinoma'.

### ACKNOWLEDGEMENTS

We thank Mr Mohamed Al-effah and Ms Lydia Kainz for excellent technical support, and Prof Dr H Denk for critical review of the manuscript.

### ABBREVIATIONS

SCC = squamous cell carcinoma, LN = lymph node, LNC = lateral neck cyst, G = histological grade

*© Cancer Research Campaign 1999*

### REFERENCES

Barr RJ (1994) The many faces of completely regressed primary melanoma. In *Malignant Melanoma and Melanocytic Lesions*. LeBoit PE (ed), pp 359. Hanley and Belfus: Philadelphia

Browder JP, Wheeler MS, Henley JT and Geratz JD (1984) Mucoepidermoid carcinoma in a cervical cyst: a case of branchiogenic carcinoma? *Laryngoscope* **94**: 107–112

Carroll WR, Zappia JJ and McClatchy KD (1993) Branchiogenic carcinoma. *J Otolaryngol* **22**: 26–28

Clark WH, Elder DE, Guerry D, Braitman LE, Trock BJ, Schultz D, Synnestvedt M and Halpern AC (1989) Model predicting survival in stage I melanoma based on tumor progression. *J Natl Cancer Inst* **81**: 1893–1904

Flanagan PM, Roland NJ and Jones AS (1994) Cervical node metastases presenting with features of branchial cysts. *J Laryngol Otol* **108**: 1068–1071

Hall SF and Dexter DF (1993) Cystic cervical metastasis are not branchiogenic carcinomas. *J Otolaryngol* **22**: 184–189

Jacobsen J, Jorgenson KF, Gosvig L and Johansen J (1998) Lymph node metastases in the neck from unknown primary. A series of 93 patients treated during a period of 20 years. *Br J Cancer* **77S**: 28

Jones AS, Cook JA, Phillips DE and Roland NJ (1993) Squamous cell carcinoma presenting as an enlarged cervical lymph node: the occult primary. *Cancer* **72**: 1756–1761

Khafif RA, Prichep R and Minkowitz S (1989) Primary branchiogenic carcinoma. *Head Neck* **11**: 153–163

Knobber D, Lobeck H and Steinkamp HJ (1995) Gibt es die malignisierte Halszyste doch? *HNO* **43**: 104–107

Martin H, Morfit HM and Ehrlich H (1950) The case for branchiogenic cancer (malignant branchioma). *Ann Surg* **132**: 867–887

Micheau C, Cachin Y and Caillou B (1974) Cystic metastases in the neck revealing occult carcinoma of the tonsil: a report of six cases. *Cancer* **33**: 228–238

Micheau C, Kijanienko J, Luboinski B and Richard J (1990) So-called branchiogenic carcinoma is actually cystic metastasis in the neck from a tonsillar primary. *Laryngoscope* **100**: 878–883

Parks SS and Karmody CS (1992) The first branchial cleft carcinoma. *Arch Otolaryngol Head Neck Surg* **118**: 969–971

Redaelli de Zinis LO, Nicolai P, Piccioni LO, Tonoli S, Vanoni V and Bignardi M (1998) Cervical metastatic carcinoma from an unknown primary: a study on 65 patients. *Br J Cancer* **77S**: 28

Regauer S, Gogg-Kamerer M, Braun H and Beham A (1997) Lateral neck cysts – the branchial theory revisited. A critical review and clinicopathological study of 97 cases with special emphasis on the cytokeratin profile. *APMIS* **105**: 623–630

Sessions RB and Hudkins CP (1993). Malignant cervical adenopathy. *Otolaryngology – Head and Neck Surgery*, 2nd edn. Cummings CW, Schuller DE (eds), pp 1605–1625. Mosby Year Book: St Louis

Shah J (ed) (1996) *Head and Neck Surgery*, 2nd edn, p 362. Mosby-Wolfe: New York

Shreedhar R and Tooley AH (1984) Carcinoma arising in a branchial cyst. *Br J Surg* **75**: 115

Sobin LH and Wittekind C (eds) (1997) *TNM Classification of Malignant Tumours*, 5th edn. Wiley-Liss: New York

Spiro RH, DeRose G and Strong EW (1983) Cervical lymph node metastasis of occult origin. *Am J Surg* **146**: 299–304

Thompson L and Heffner DK (1998) The clinical importance of cystic squamous cell carcinoma in the neck. A study of 136 cases. *Cancer* **82**: 944–956

von Volkmann R (1882) Das tiefe branchiogene Halskarzinom. *Zentralblatt Chirurgie* **9**: 49–63

Wolff M, Rankow R and Fliegel J (1979) Branchiogenic carcinoma – fact or fallacy? *J Maxillofacial Surg* **7**: 41–47

*© Cancer Research Campaign 1999*
